# Prenatal diagnosis and molecular cytogenetic analyses of a paternal inherited deletion of 1q23.3 encompassing *PBX1* gene

**DOI:** 10.1186/s13039-022-00632-y

**Published:** 2022-12-21

**Authors:** Man Luo, Xia Gu, Ting Zhou, Chaoli Chen

**Affiliations:** 1 Department of Obstetrics, Maternal and Child Health Hospital of Hubei Province, Wuhan, Hubei People’s Republic of China; 2 Medical Genetics Center, Maternal and Child Health Hospital of Hubei Province, Wuhan, Hubei People’s Republic of China

**Keywords:** *PBX1*, Chromosomal microdeletions/microduplications, Chromosomal microarray analysis, Congenital anomalies of kidney and urinary tract, Ultrasound, Prenatal diagnosis

## Abstract

**Background:**

Patients with deletions involving the long arm of chromosome 1 are rare. The *PBX1* gene is located on chromosome 1q23.3. *PBX1* encodes a transcription factor which promotes protein–protein interaction and plays a crucial role in several developmental processes. *PBX1* haploinsufficiency had been reported to lead syndromic congenital anomalies of kidney and urinary tract (CAKUT) in humans.

**Case presentation:**

In this research, a 24-year-old woman (gravida 1, para 0) underwent amniocentesis at 22 weeks’ gestation because of a horseshoe kidney of the fetus on prenatal ultrasound.

**Results:**

Chromosomal microarray analysis (CMA) from this family revealed a 1.14 Mb paternal inherited deletion on chromosome 1q23.3, spanning from position 163,620,000 to 164,760,000 (hg19). Trio whole-exome sequencing (WES) showed heterozygous deletions in exons 1–2 of the *PBX1* in fetal and paternal samples. At the 3-year follow-up, the baby did not have an abnormal phenotype except a horseshoe kidney.

**Conclusion:**

We provide a detailed description of the phenotype in a family with paternal inherited deletion of 1q23.3 encompassing exons 1–2 of the *PBX1* gene. Combination of karyotype analysis, CMA, WES, prenatal ultrasound and genetic counseling is helpful for the prenatal diagnosis of chromosomal microdeletions/microduplications.

## Introduction

The incidence of chromosome 1q deletion in the population has not been reported due to the limited number of reported cases. Available data on the patients with the deletions on chromosome 1q, indicate that the most common clinical features include palmprint abnormality, fingernail dysplasia, abnormal ears, microcephaly, intellectual disability, fetal growth restriction, short limbs, congenital anomalies of kidney and urinary tract (CAKUT) and external genital malformations [1].

CAKUT are common finding on fetal ultrasound, accounting for 20–30% of birth defects, present in 3–7 out of 1000 births [2]. CAKUT is the most common cause of end stage renal disease in children, leading to high mortality and morbidity in these patients [3]. Though the etiology of most cases is unknown, multiple lines of evidence suggest a strong contribution of genetic defects, such as some monogenic mutations and copy number variations (CNVs).

The *PBX1* gene is located on chromosome 1q23.3. Recently, multiple studies demonstrated association of *PBX1* haploinsufficiency with syndromic CAKUT [4]. However, little is known about the prenatal phenotype caused by *PBX1* defects.

Here, we provide a detailed description of the phenotype and mechanisms of a family with paternal inherited deletion on chromosome 1q23.3.

## Methods

### Patients and samples

**Fig. 1:**
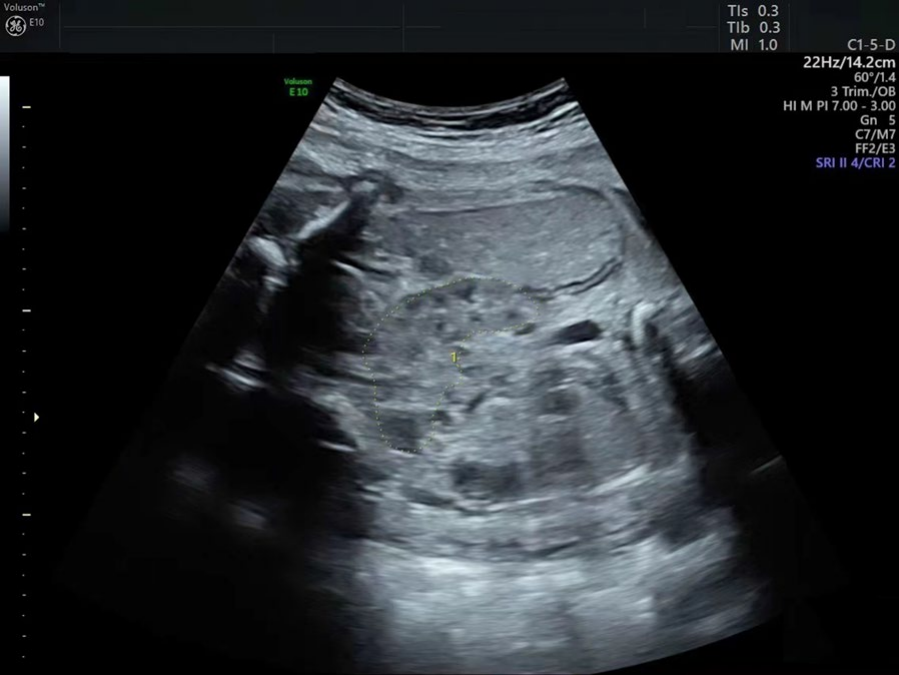
Ultrasound image of the horseshoe kidney

A 24-year-old woman (gravida 1, para 0) underwent amniocentesis at 22 weeks’ gestation because of horseshoe kidney of the fetus on prenatal ultrasound (Fig. 1). She and her 25-year-old husband were normal, healthy and non-consanguineous. There was no family history of birth defects or genetic diseases.

- GTG-banding karyotype analysis was performed on cultured amniocytes and parental blood samples. CMA on uncultured amniocytes and parental blood samples was performed using the Affymetrix CytoScan 750 K chip, which includes 550k non-polymorphic markers and 200k SNP markers [5].
- We performed Trio whole-exome sequencing (WES) on the family. The Novaseq6000 platform (Illumina, San Diego, USA), with 150 bp pair-end sequencing mode, was used for sequencing the genomic DNA of the family. The sequencing reads were aligned to the human reference genome (hg38/GRCh38) using the Burrows-Wheeler Aligner tool [6].

## Results

**Fig. 2:**
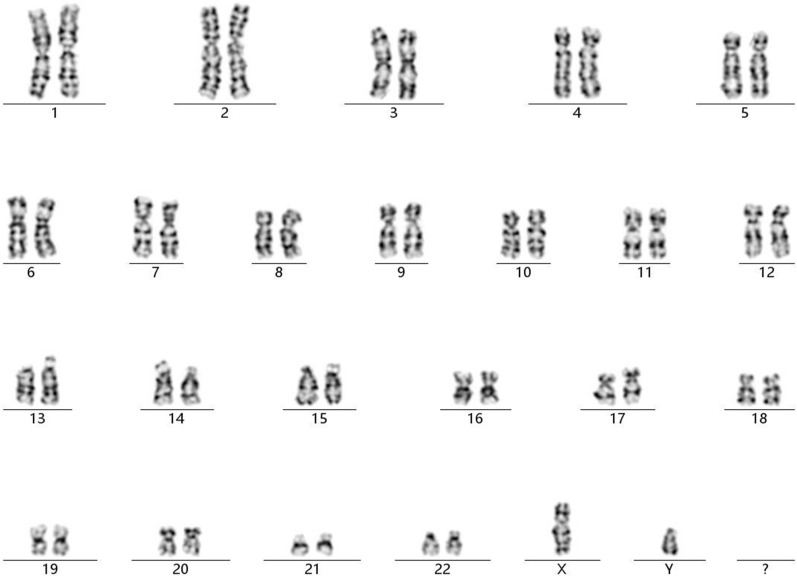
The karyotype of 46,XY

**Fig. 3:**
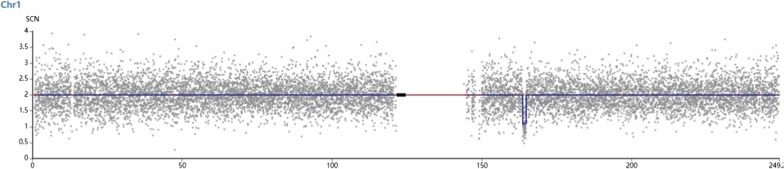
CMA detected a 1.14-Mb chromosomal deletion in the region of 1q23.3 (arr[GRCh37]1q23.3(163,620,000_164,760,000)x1)

Chromosomal GTG-banding revealed a karyotype of 46,XY (Fig. 2). CMA detected a 1.14-Mb chromosomal deletion in the region of 1q23.3, which is to be reported according to International System of Cytogenomic Nomenclature 2020 (ISCN 2020) [7] as arr[GRCh37] 1q23.3(163,620,000_164,760,000)x1 (Fig. 3). Then we performed both CMA and chromosomal GTG-banding using the samples from the parents’ peripheral blood. Their karyotypes were normal. The CMA results showed the father had a 1.14-Mb chromosomal deletion like the fetus. We performed a comprehensive physical examination of the parents and failed to identify anything abnormal.

**Fig. 4:**
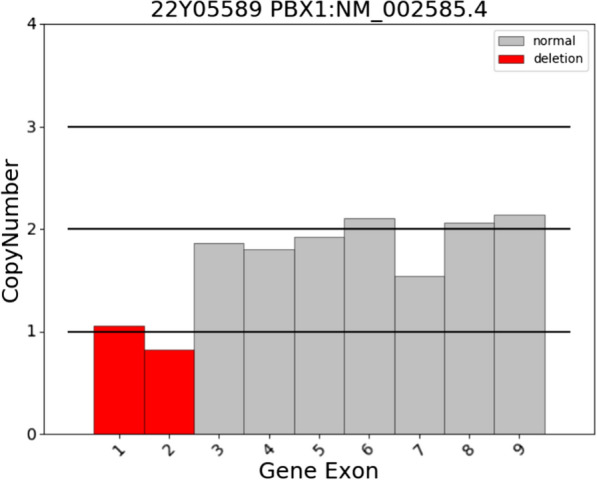
Trio-WES showed heterozygous deletions in exons 1–2 of the *PBX1* gene

Trio-WES on the family showed no pathogenic SNV and InDel variants related to the phenotype of this case were detected in the sample of the subjects, but heterozygous deletions in exons 1–2 of the *PBX1* gene were detected in fetal and paternal samples (Fig. 4).

Ultrasound examination showed no intrauterine growth restriction (IUGR) or dysmorphisms (except horseshoe kidney) in the fetus. Considering the father himself is a carrier of chromosome 1q23.3 deletion, all of his children have a one in two chance of inheriting this deletion, after genetic counseling, the parents decided to continue the pregnancy.

At 40 weeks of gestation, the expectant mother gave birth vaginally to a male baby. The baby’s growth parameters at birth were in the normal ranges. Apgar scores were 9/9/10. The baby received a complete physical examination and the results were normal (except horseshoe kidney). At 36-month checkup, the baby was developing normally (Intelligence Quotient, IQ = 109).

## Discussion

*PBX1* encodes a transcription factor which promotes protein-protein interaction and plays a crucial role in several developmental processes. In human, *PBX1* is constitutively expressed in human bone-derived cells (HBDC) and is strongly expressed in fetal kidneys and brain [8].

The deletions of chromosome 1q described by conventional cytogenetic techniques had showed that patients presented abnormalities of kidney and urinary tract, microbrachycephaly, developmental delay and hand anomalies [9].

With molecular cytogenetic techniques especially CMA, some of patients harboring microdeletions with precise breakpoints were reported, which offered the opportunity to identify *PBX1* as a promising candidate gene associated with renal malformation [10].

In 2017, Le Tanno et al. reported several de novo microdeletions at 1q23.3-q24.1 locus. Among of these patients, the smallest overlapping region (SRO) focus on *PBX1* gene, which is proposed to be relevant to syndromic CAKUT [8]; In addition, Laurence et al. identified five de novo heterozygous loss of function mutations in *PBX1* gene or microdeletions involving the *PBX1* gene in 204 unrelated CAKUT patients [11]. Based on these findings, it provides convincing evidence that *PBX1* gene causes CAKUT by haploinsufficiency mechanism.

Besides the heterozygous loss or microdeletions involving the *PBX1* gene, autosomal dominant (de novo) mutations in *PBX1* are known to cause congenital abnormalities of the kidney and urinary tract (CAKUT), with or without extra-renal abnormalities [12], amplification of chromosome 1q23.3 is associated with urothelial carcinoma [13].

Patients with pathogenic *PBX1* variants/microdeletions showed pleiotropic developmental defects, including external ear anomalies, abnormal branchial arch derivatives, heart malformations, diaphragmatic hernia, renal hypoplasia and ambiguous genitalia [4, 8, 11, 14]. Developmental delays and craniofacial dysmorphy were also reported in patients who carried *PBX1* gene mutations or deletions.

*PBX1* could be a candidate gene for fetal growth restriction, renal hypoplasia and congenital heart disease.

CMA of this fetus revealed a 1.14 Mb paternal inherited deletion on chromosome 1q23.3, Trio-WES on the family showed no pathogenic SNV and InDel variants related to the phenotype of this case were detected in the sample of the subjects, but heterozygous deletions in exons 1–2 of the *PBX1* gene were detected in fetal and paternal samples.

## Conclusion

In conclusion, we provide a detailed description of the phenotype in a family with paternal inherited deletion of 1q23.3 encompassing exons 1–2 of the *PBX1* gene. The heterozygous deletions in exons 1–2 of *PBX1* resulted in the fetus with a horseshoe kidney, but the same deletion had no phenotype in the father. More studies is needed to provide further insights into the pathogenesis of 1q23.3 deletion.

Combination of karyotype analysis, CMA, WES, prenatal ultrasound and genetic counseling is helpful for the prenatal diagnosis of chromosomal microdeletions/microduplications.

## Data Availability

Please contact the corresponding author for data requests.
